# Supplementary material for: Cell Stress Induces Mislocalization of Transcription Factors with Mitochondrial Enrichment
Source: Int J Mol Sci. 2021 Aug 17;22(16):8853. doi: 10.3390/ijms22168853 (PMC8396249; doi:10.3390/ijms22168853)
Supplement: Supplementary file 1 [file ijms-22-08853-s001.zip › ijms-1311235-supplementary.pdf]

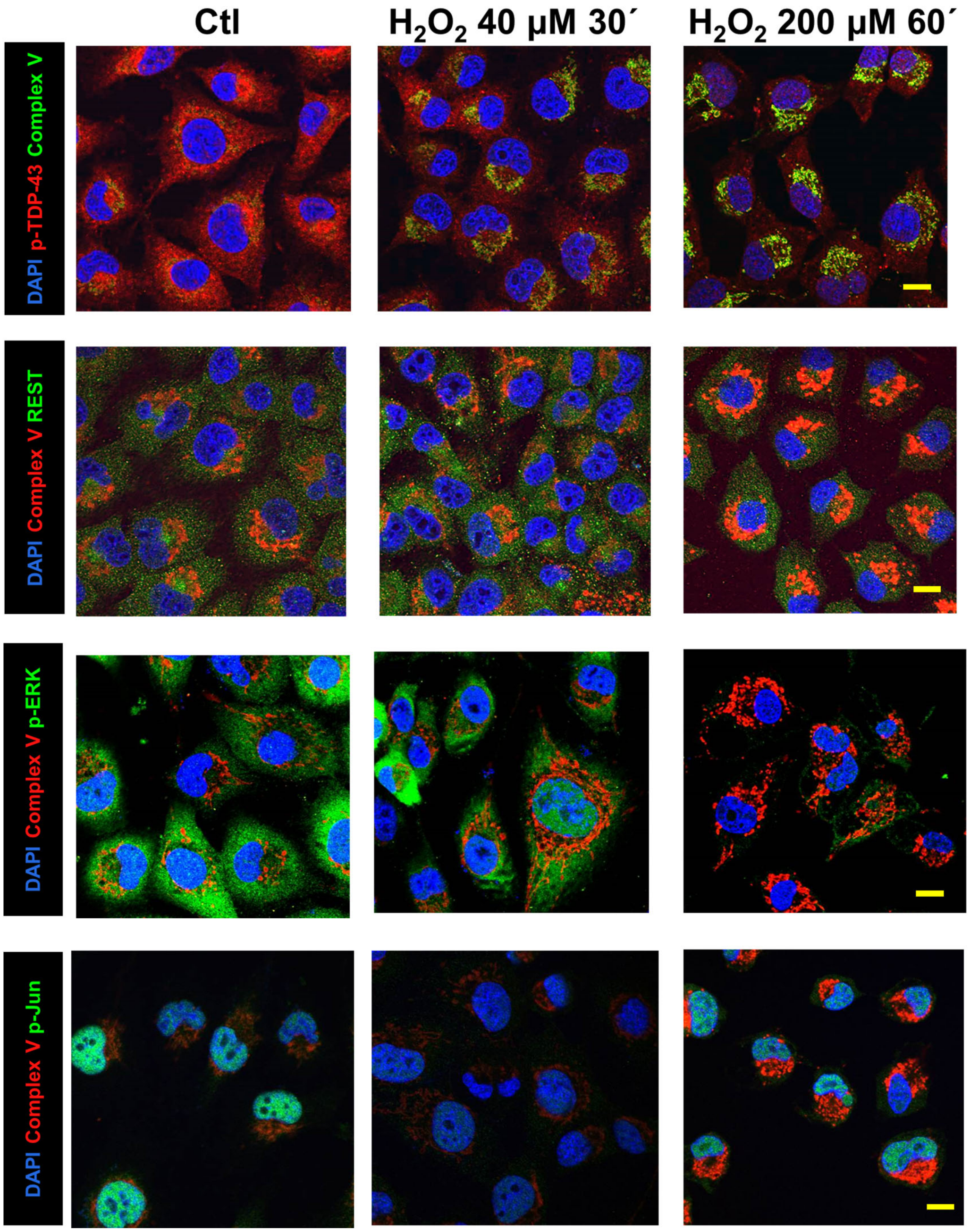

Supplemental Figure S1. Higher magnification images of confocal microscopy present in Figure 1. Scale bars shown are 20 micrometer long.

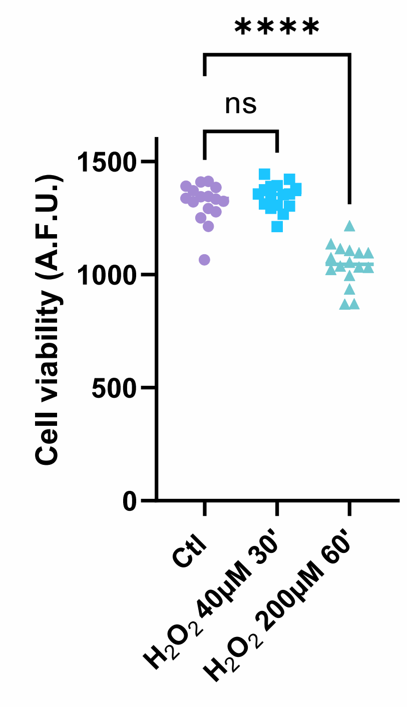

Supplemental Figure S2. Oxidative stress induces changes in cell viability at highest concentrations employed. Data shown is for independent cell plates. **** indicates p<0.0001 by Uncorrected Fisher's LSD post-hoc test after one-way ANOVA.

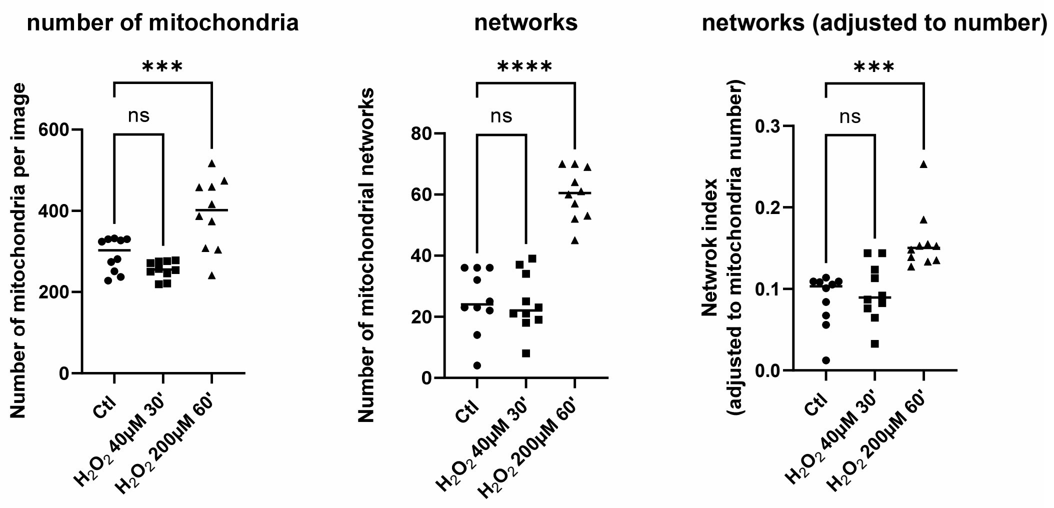

Supplemental Figure S3. Oxidative stress induces changes in mitochondrial number and networks. Data shown is for 9 independent cell plates. **** indicates p<0.0001 and *** p<0.001 by Uncorrected Fisher's LSD post-hoc test after one-way ANOVA

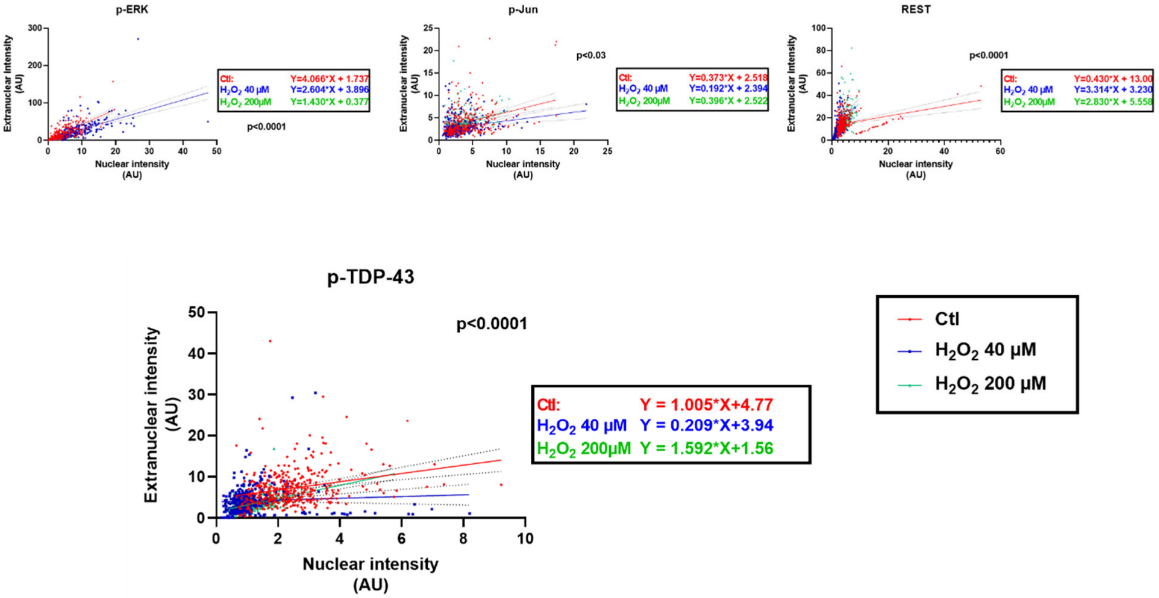

Supplemental Figure S4. Oxidative stress induces changes between the nucleocytosolic relationships of proteins implicated in neurodegeneration. In all cases, total immunoreactivities found in nuclear and cytosolic compartments are related linearly significantly (in all cases $p<0.001$), though the slope is significantly affected by oxidative stress (shown p values for comparison of slopes). Shown are the linear relationships between nuclear and cytosolic content, with 95% confidence intervals indicated with discontinous lines (n=200 to 296 cells for p-TDP-43; n=191-255 for p-ERK; n=234-326 for p-Jun and n=217-415 for REST, obtained in at least 4 independent replicates). Inset of graphs show the equations of the linear relationships, separated by colors.

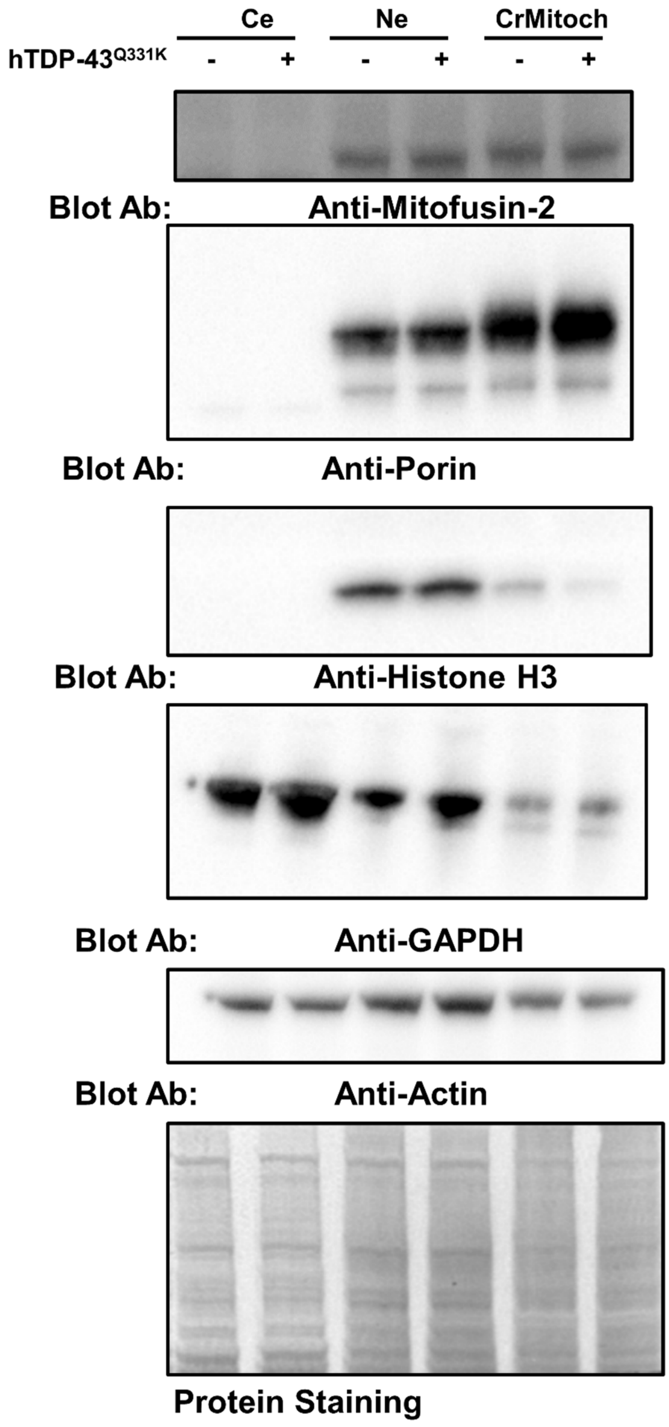

Supplemental Figure S5. Enrichment of protein markers in subcellullar fractionation. As shown by western-blot analyses of brain lysates after subcellular fractionation, in addition to nuclear enriched (Ne) and cytosolic enriched (Ce) compartments, crude mitochondrial fractions (CrMitoch) both non transgenic and transgenic hTDP-43 mice show the relative enrichment of Mitofusin-2 and porin in crude mitocondria, with almost the absence of histone H3 in non-nuclear fractions, and the high abundance of GAPDH in cytosolic extracts. Actin was distributed equally among the three fractions.

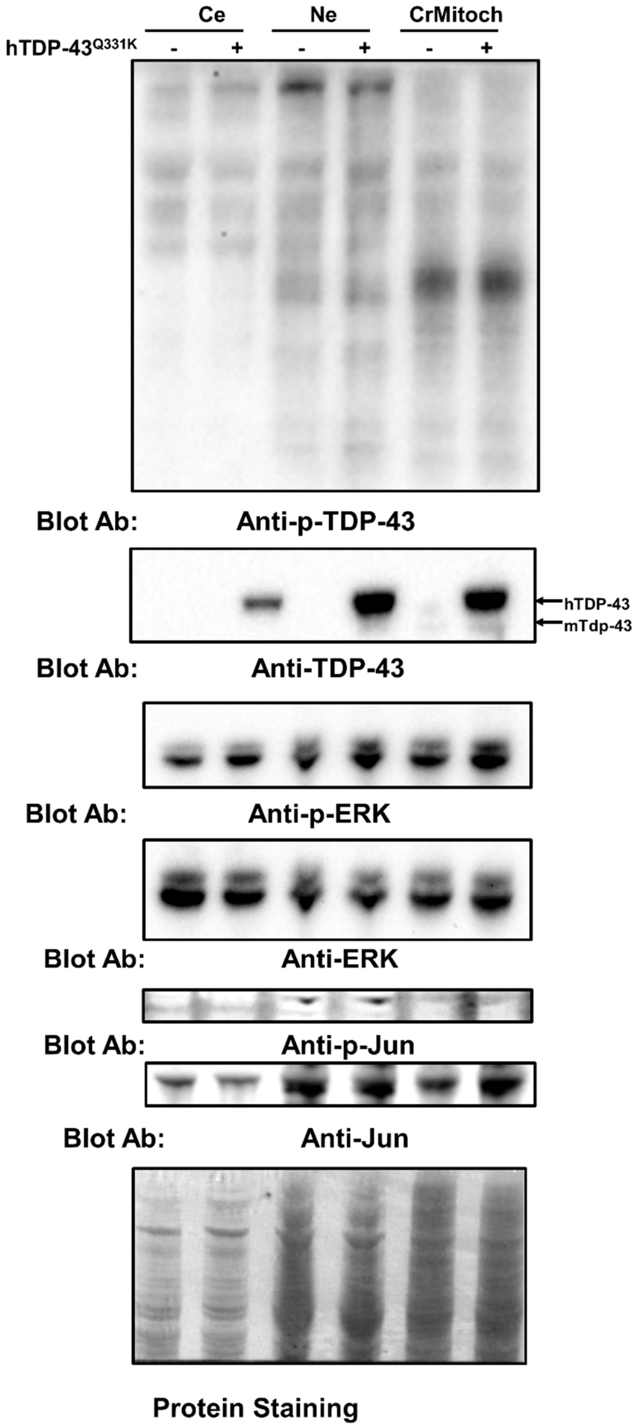

Supplemental Figure S6. Cellular subfractionation evidence for in vivo colocalization of proteins implicated in neurodegeneration with mitochondrial components. As shown by western-blot analyses of brain lysates after subcellular fractionation, in addition to nuclear enriched (Ne) and cytosolic enriched (Ce) compartments, crude mitochondrial fractions (CrMitoch) both non transgenic and transgenic hTDP-43 mice show the presence of p-TDP-43, p-ERK and Jun. Levels were quantified by densitometry in brains from 90 day old mice. Western-blot shown are for male specimens.

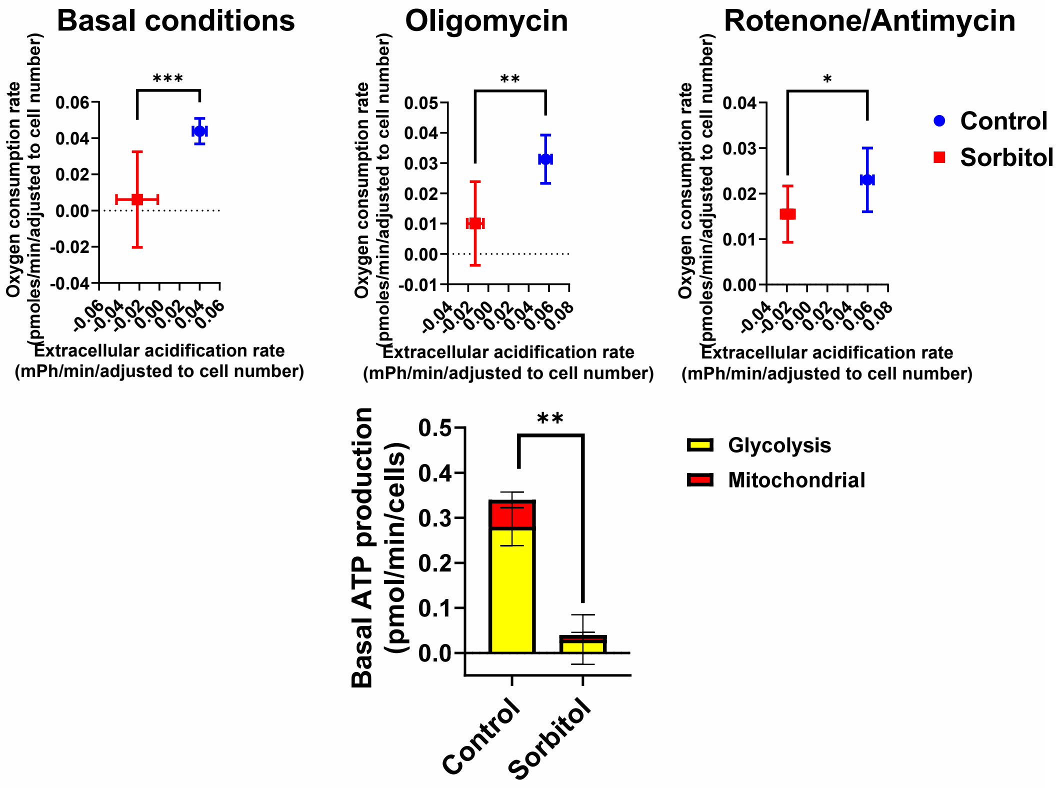

Supplemental figure S7. TDP-43 aggregation is linked to mitochondrial dysfunction. Sorbitol incubation in SHSY-5Y stress induces changes in oxygen consumption and extracellular acidification rates, measured by using Seahorse respirometry. Data shown is for 6 independent cell plates for each condition. *** indicates p<0.001, ** p<0.01 and * p<0.05 by Uncorrected Fisher's LSD post-hoc test after one-way ANOVA

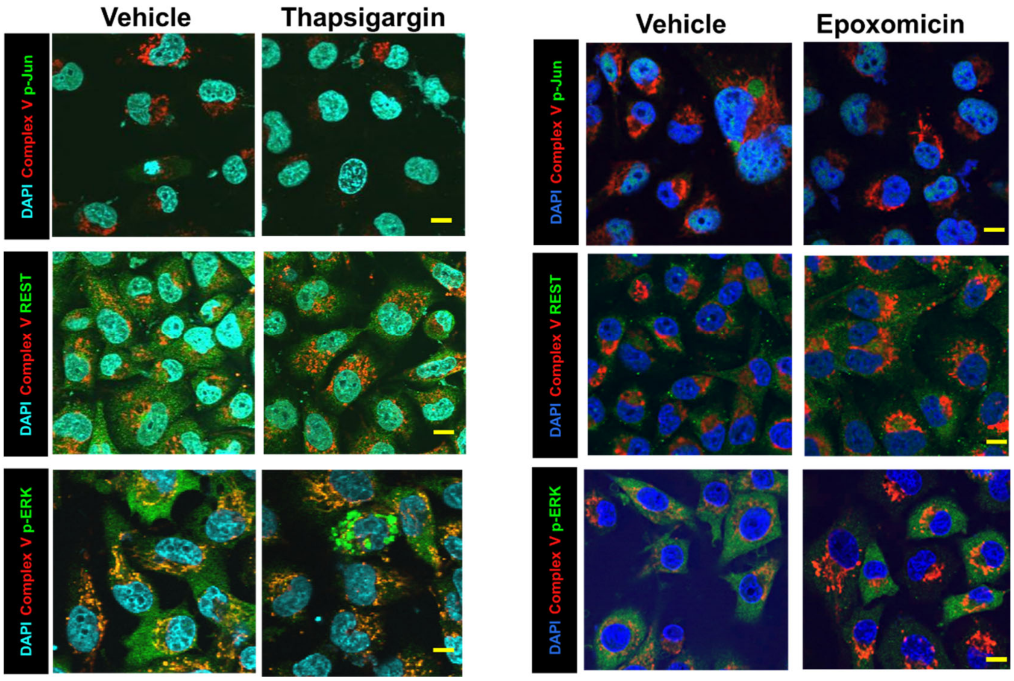

Supplemental Figure S8. Higher magnification images of confocal microscopy present in Figures 5 and 6. Scale bars shown are 20 micrometer long.

Supplemental Table S1. Effect of mutated *TARDBP* overexpression in subcellular distribution of transcription factors

Source of Variation	% of total variation TDP-43	% of total variation p-TDP-43	% of total variation p-TDP-43/TDP-43	% of total variation ERK	% of total variation p-ERK	% of total variation p-ERK/ERK	% of total variation Jun	%of total variation p-Jun	%of total variation p-Jun/Jun	% of total variation REST
Subcellular location	8.455	47.63****	5.106	20.96	52.46****	42.86***	16.76*	63.03****	15.16	39.96**
Sex	8.736	13.62**	4.433	0.8403	0.5431	1.015	3.633	9.650***	0.1989	0.6926
hTDP-43 overexpression	22.51**	1.437	14.30*	0.7036	3.221	3.696	3.566	0.01932	5.320	0.8719
Subcellular location x Sex	2.034	1.860	4.662	1.007	13.81**	3.210	9.362	6.350*	4.894	0.8915
Subcellular location x hTDP-43 overexpression	4.407	7.622	2.430	0.08271	0.9421	0.8392	9.667	1.460	7.719	2.031
Sex x hTDP-43 overexpression	4.588	2.278	5.143	0.03548	0.7200	0.6995	3.620	0.3800	2.074	0.004417
Subcellular location x Sex x hTDP-43 overexpression	0.7420	0.5555	3.948	0.3125	0.2751	0.2329	8.345	4.474*	3.752	0.2244

****Indicate $p<0.0001$, *** $p<0.001$, ** $p<0.01$, and $p<0.05$ after three way ANOVA

Supplemental Table S2. Effect of H_2O_2 treatment in protein subcellular distribution by confocal microscopy

Source of Variation	% of total variation TDP-43	% of total variation p-Jun	% of total variation p-ERK	% of total variation REST
Interaction	7.862****	0.3051	4.401****	1.407****
Stress	12.23****	3.610***	12.31****	4.307****
Location	31.14****	0.04323	12.04****	39.41****

****Indicate $p<0.0001$ after two way ANOVA

Supplemental Table S3. Effect of Epoxomycin treatment in protein subcellular distribution by confocal microscopy

Source of Variation	% of total variation p-ERK	% of total variation p-Jun	% of total variation REST
time	0.02292****	0.1592****	0.1421**
Cytosol vs nuclei	90.56****	31.72****	32.14****
Epoxomycin treatment	7.383****	2.957****	0.0002409
time x Cytosol vs nuclei	0.2466****	5.703****	0.1810***
time x Epoxomycin treatment	0.3134****	1.444****	1.139****
Cytosol vs nuclei x Epoxomycin treatment	2.433****	51.37****	0.3210***
time x Cytosol vs nuclei x Epoxomycin treatment	0.4035****	1.042****	0.02023

****Indicate $p<0.0001$ after three way ANOVA

Supplemental Table S4. Effect of thapsigargin treatment in protein subcellular distribution by confocal microscopy

Source of Variation	% of total variation p-ERK	% of total variation p-Jun	% of total variation REST
Time	0.04933****	1.269****	3.619****
Cytosol vs nuclei	79.41****	91.46****	22.06****
Thapsig treatment	12.88****	1.709****	2.153****
Time x Cytosol vs nuclei	0.08946****	1.512****	0.3063****
Time x Thapsig treatment	1.417****	2.077****	1.651****
Cytosol vs nuclei x Thapsig treatment	3.749****	0.5553****	0.7645****
Time x Cytosol vs nuclei x Thapsig treatment	1.006****	0.5563****	0.8305****

****Indicate $p<0.0001$ after three way ANOVA

Supplemental Table S5. Primers employed for quantitation of REST transcriptional regulation.

**Gene**	**Accession No.**	**Application**	**Sequence (5'→ 3')**
BID	NM_197967	RT-PCR	(F) agtgggagggctacgatgag
			(R) gatgctacggtccatgctgt
PUMA	NM_014417	RT-PCR	(F) cccgtgaagagcaaatgag
			(R) accccctgatgaaggtgag
BAX	NM_138761	RT-PCR	(F) tctgacggcaacttcaactg
			(R) cgtcccaaagtaggagagga
FADD	NM_003824	RT-PCR	(F) ctggggaagaagacctgtg
			(R) gcacacgctctgtcaggtt
DAXX	NM_001350	RT-PCR	(F) aagcctccttggattctggt
			(R) atcatcctcctgaccctcct
FAS	NM_000043	RT-PCR	(F) agttggggaagctctttcactt
			(R) cagtcttcctcaattccaatcc
PSEN2	NM_000447	RT-PCR	(F) cctcggggacttcatcttc
			(R) tgaacacagcaagcagcag
PESENEN	NM_172341	RT-PCR	(F) tgaacctggagcgagtgtc
			(R) taggctgggacaaggaagg
P35	NM_003885	RT-PCR	(F) caaaccaggagcattttgtgt
			(R) attcctgtggcttgttctgtg
P39	NM_003936	RT-PCR	(F) ccttcattacgcctgcaaa
			(R) tctcgttgcccatgtagga

KCNQ2	NM_172106	RT-PCR	(F) gcgcaacgccttctacc
			(R) gacagcacgaggcagga
KV2.1	NM_004975	RT-PCR	(F) ggaaggcgaggagttcg
			(R) gggcaatggtggagagg
KCNJ6	NM_002240	RT-PCR	(F) ctctcggtgctgatgtgaaa
			(R) tgaaacggagcaagactgaa
CAT	NM_001752	RT-PCR	(F) atccagaagaaagcggtcaa
			(R) cagatttgccttctcccttg
FOXO1	NM_002015	RT-PCR	(F) tggggcaacctgtcctac
			(R) ggcacgctcttgaccatc
SOD1	NM_000454	RT-PCR	(F) ggcaaaggtggaaatgaaga
			(R) gggcctcagactacatccaa
1433ζ	NM_145690	RT-PCR	(F) agcccgtaggtcatcttgg
			(R) tgaagcattggggatcaag
ARC	NM_015193	RT-PCR	(F) cgcctggagaagaatcagag
			(R) gggaaccttgagacctgttg
BCL2	NM_000633	RT-PCR	(F) ggaggattgtggccttcttt
			(R) gccgtacagttccacaaagg
CASP2	NM_032982	RT-PCR	(F) ttgccgaagatgagactgc
			(R) gcgttcaccttaaccagca
ANT1	NM_001151	RT-PCR	(F) gggctctaccagggtttca
			(R) cgtcacactctgggcaatc
PDCD7	NM_005707	RT-PCR	(F) gcaggaggtggaggagaag
			(R) tggaggacagaccccctttc
MAPK11	NM_002751	RT-PCR	(F) taccggcaggagctgaac
			(R) ttcttcaccgccaccttc
MAPK12	NM_002969	RT-PCR	(F) ccaccttcaccttccacct
			(R) gcgtctgctctgatggatg
GAP43	NM_001130064	RT-PCR	(F) gggaggcttgaggaaaaatc
			(R) gcagcttggacatcatcctt
EGR1	NM_001964	RT-PCR	(F) gttaccccagccaaaccac
			(R) tgggttggtcatgctcact
NRXN3	NM_004796	RT-PCR	(F) gggaacaacacagacgacct
			(R) ctcggctcacattcaacaaa
GRIA4	NM_000829	RT-PCR	(F) gcagcgccttacatatctcc
			(R) ccaaccattttgtcctgctt
MEF2C	NM_001193350	RT-PCR	(F) ggggactatggggagaaaaa
			(R) gcttgttggtgctgttgaag
SANP25	NM_003081	RT-PCR	(F) tcatccgcagggtaacaaa
			(R) ttggcctcatcaattctgg
SST	NM_001048	RT-PCR	(F) gaccccagactccgtcagt
			(R) gctcaagcctcatttcatcc
ATP2B2	NM_001683	RT-PCR	(F) ggctcacacagaaggaggag
			(R) ggatggaggttcgagattca
GAD1	NM_000817	RT-PCR	(F) ttgcaccagtgtttgtcctc
			(R) aggaccagtttaggcacagc
GAD2	NM_000818	RT-PCR	(F) gacctgctccagtctccaaa

			(R) agggcgcacagtttgtttc
CYCS	NM_018947	RT-PCR	(F) tgaaaagggaggcaagca
			(R) ccccagatgatgcctttg
mCYCS	NM_007808	RT-PCR	(F) ccaaatctccacggtctgtt
			(R) gtctgccctttctcccttct
mMAPK11	NM_011161	RT-PCR	(F) ccagaaggtggctgtaaagaag
			(R) gcctgacacttgacgatgttatt
mCASP3	NM_00981	RT-PCR	(F) tgtcatctcgctctggtacg
			(R) aaatgaccccttcatcacca
mβ-actin	NM_007393	RT-PCR	(F) tgggacgacatggagaaga
			(R) tggggtgttgaaggtctca
mCIDEA	NM_007702	RT-PCR	(F) agggacaacacgcatttca
			(R)cattgagacagccgaggaa
CALB1	NM_004929	RT-PCR	(F) gaactctggaggaacgctga
			(R) aggctgtgatgagggatgac
SCN3B	NM_018400	RT-PCR	(F) attgtttcccctggcttctc
			(R) gcctccacctcctctctctt
GABRB3	NM_021912	RT-PCR	(F) gcttcttggcttctctggtg
			(R) aacgagatgccattcactcc
JIP1	NM_005456	RT-PCR	(F) caccacgctcaacctctttc
			(R) gtgtctgctcccctgtcttc

p-Jun

**Source of Variation**	**% of total variation p-Jun**	**P value**
Time	0.1592****	<0.0001
Cytsol vs nuclei	31.72****	<0.0001
Epoxomyc	2.957****	<0.0001
Time x Cytsol vs nuclei	5.703****	<0.0001
Time x Epoxomyc	1.444****	<0.0001
Cytsol vs nuclei x Epoxomyc	51.37****	<0.0001
Time x Cytsol vs nuclei x Epoxomyc	1.042****	<0.0001

REST

**Source of Variation**	**% of total variation**	**P value**
Time	0.1421**	0.0128
Cytosol vs Nucli	32.14****	<0.0001
Epoxom treat	0.0002409	0.9183
Time x Cytosol vs Nucli	0.1810***	0.0050
Time x Epoxom treat	1.139****	<0.0001
Cytosol vs Nucli x Epoxom treat	0.3210***	0.0002
Time x Cytosol vs Nucli x Epoxom treat	0.02023	0.3474

Effect of Thapsigargin treatment in protein subcellular distribution by confocal microscopy

Source of Variation	% of total variation p-ERK	% of total variation p-Jun	% of total variation REST
Time	0.04933****	1.269****	3.619****
Cytosol vs nuclei	79.41****	91.46****	22.06****
Thapsig treatment	12.88****	1.709****	2.153****
Time x Cytosol vs nuclei	0.08946****	1.512****	0.3063****
Time x Thapsig treatment	1.417****	2.077****	1.651****
Cytosol vs nuclei x Thapsig treatment	3.749****	0.5553****	0.7645****
Time x Cytosol vs nuclei x Thapsig treatment	1.006****	0.5563****	0.8305****

p-Jun

Source of Variation	% of total variation	P value
Time	1.269****	<0.0001
cytosol nuclei	91.46****	<0.0001
thapsi	1.709****	<0.0001
time x cytosol nuclei	1.512****	<0.0001
time x thapsi	2.077****	<0.0001
cytosol nuclei x thapsi	0.5553****	<0.0001
time x cytosol nuclei x thapsi	0.5563****	<0.0001

REST

Source of Variation	% of total variation	P value
Time	3.619****	<0.0001
Nuclei vs cytosol	22.06****	<0.0001
Thapsigargin treatment	2.153****	<0.0001
Time x Nuclei vs cytosol	0.3063****	0.0001
Time x Thapsigargin treatment	1.651****	<0.0001
Nuclei vs cytosol x Thapsigargin treatment	0.7645****	<0.0001
Time x Nuclei vs cytosol x Thapsigargin treatment	0.8305****	<0.0001

Effect of H_2O_2 treatment in protein subcellular distribution by confocal microscopy

Source of Variation	% of total variation TDP-43	% of total variation p-Jun	% of total variation p-ERK	% of total variation REST
Interaction	7.862****	0.3051	4.401****	1.407****
Stress	12.23****	3.610***	12.31****	4.307****
Location	31.14****	0.04323	12.04****	39.41****

Table Analyzed p-jun

**Source of Variation p-Jun**	**% of total variation**	**P value**
Interaction	0.3051	0.0634
Stress	3.610***	<0.0001
Location	0.04323	0.3763

**Source of Variation p-ERK**	**% of total variation**	**P value**
Interaction	4.401****	<0.0001
Stress	12.31****	<0.0001
Location	12.04****	<0.0001

Source of Variation REST	% of total variation	P value
Interaction	1.407****	<0.0001
Stress	4.307****	<0.0001
Location	39.41****	<0.0001

Effect of mutated TARDBP overexpression in subcellular distribution of transcription factors

Source of Variation	% of total variation TDP-43	% of total variation p-TDP-43	% of total variation p-TDP-43/TDP-43	% of total variation ERK	% of total variation p-ERK	% of total variation p-ERK/ERK	% of total variation Jun	%of total variation p-Jun	%of total variation p-Jun/Jun	% of total variation REST
Subcellular location	8.455	47.63****	5.106	20.96	52.46****	42.86***	16.76*	63.03****	15.16	39.96**
Sex	8.736	13.62**	4.433	0.8403	0.5431	1.015	3.633	9.650***	0.1989	0.6926
hTDP-43 overexpression	22.51**	1.437	14.30*	0.7036	3.221	3.696	3.566	0.01932	5.320	0.8719
Subcellular location x Sex	2.034	1.860	4.662	1.007	13.81**	3.210	9.362	6.350*	4.894	0.8915
Subcellular location x hTDP-43 overexpression	4.407	7.622	2.430	0.08271	0.9421	0.8392	9.667	1.460	7.719	2.031
Sex x hTDP-43 overexpression	4.588	2.278	5.143	0.03548	0.7200	0.6995	3.620	0.3800	2.074	0.004417
Subcellular location x Sex x hTDP-43 overexpression	0.7420	0.5555	3.948	0.3125	0.2751	0.2329	8.345	4.474*	3.752	0.2244
